# There is more than one way to turn a spherical cellular monolayer inside out: type B embryo inversion in *Volvox globator*

**DOI:** 10.1186/1741-7007-9-89

**Published:** 2011-12-29

**Authors:** Stephanie Höhn, Armin Hallmann

**Affiliations:** 1 Department of Cellular and Developmental Biology of Plants, University of Bielefeld, Universitätsstrasse 25, 33615 Bielefeld, Germany

## Abstract

**Background:**

Epithelial folding is a common morphogenetic process during the development of multicellular organisms. In metazoans, the biological and biomechanical processes that underlie such three-dimensional (3D) developmental events are usually complex and difficult to investigate. Spheroidal green algae of the genus *Volvox *are uniquely suited as model systems for studying the basic principles of epithelial folding. *Volvox *embryos begin life inside out and then must turn their spherical cell monolayer outside in to achieve their adult configuration; this process is called 'inversion.' There are two fundamentally different sequences of inversion processes in Volvocaceae: type A and type B. Type A inversion is well studied, but not much is known about type B inversion. How does the embryo of a typical type B inverter, *V. globator*, turn itself inside out?

**Results:**

In this study, we investigated the type B inversion of *V. globator *embryos and focused on the major movement patterns of the cellular monolayer, cell shape changes and changes in the localization of cytoplasmic bridges (CBs) connecting the cells. Isolated intact, sectioned and fragmented embryos were analyzed throughout the inversion process using light microscopy, confocal laser scanning microscopy, scanning electron microscopy and transmission electron microscopy techniques. We generated 3D models of the identified cell shapes, including the localizations of CBs. We show how concerted cell-shape changes and concerted changes in the position of cells relative to the CB system cause cell layer movements and turn the spherical cell monolayer inside out. The type B inversion of *V. globator *is compared to the type A inversion in *V. carteri*.

**Conclusions:**

Concerted, spatially and temporally coordinated changes in cellular shapes in conjunction with concerted migration of cells relative to the CB system are the causes of type B inversion in *V. globator*. Despite significant similarities between type A and type B inverters, differences exist in almost all details of the inversion process, suggesting analogous inversion processes that arose through parallel evolution. Based on our results and due to the cellular biomechanical implications of the involved tensile and compressive forces, we developed a global mechanistic scenario that predicts epithelial folding during embryonic inversion in *V. globator*.

## Background

One of the most fascinating problems in developmental biology is how embryonic cells work together to produce highly organized multicellular organisms. Although embryonic tissues may ultimately be shaped in a species-specific manner, there are basic remodeling events that are shared, even among distantly related species. Epithelial folding is one of these common morphogenetic processes and is involved in metazoan developmental stages including gastrulation, neurulation and organogenesis. Epithelia are composed of closely arranged epithelial cells that adhere together at intercellular junctions that give the epithelial sheet mechanical strength. This adherence of the cells is thought to be the key for the transmission of forces between neighboring cells through the links between the actomyosin cytoskeleton and the adherens junctions [1]. In metazoan development, folding or bending of the cell sheet is achieved by cells at the bend points that change from a cuboidal to a wedge shape. This cell wedging is observed in several invaginations, including blastopore groove formation and neurulation in amphibians and the formation of the primitive groove in birds [1-7]. It also exists in other cases, such as gastrulation in some echinoderms [8], but is less obvious in these cases, likely because mechanisms other than cell wedging are involved [1]. The bending and folding of a sheet of cells is fundamentally a biomechanical, supercellular process involving coordinated changes in the shapes of many neighboring cells that interact; however, the biological and biomechanical processes that underlie such three-dimensional (3D) morphogenetic events in metazoans are usually complex and difficult to investigate [2-7,9-19].

In the simple multicellular green algae *Volvox*, a morphogenetic process known as inversion occurs during embryogenesis. *Volvox *inversion bears significant similarity to metazoan gastrulation and has been suggested as a model system for studying the curling of an epithelium [20-25]. The similarities between the morphogenetic process of inversion in *Volvox carteri *and epithelial folding events in metazoans include cell adherence, cell shape changes, cell movements, transmission of forces between neighboring cells and the involvement of cytoskeletal elements [20,22-24,26-29]. However, embryonic *Volvox *cells have no adherens junctions; instead, they adhere together through a network of numerous and quite robust cytoplasmic bridges (CBs) that are the result of incomplete cytokinesis [22] and allow for force transmission. As in metazoans, folding or bending of the embryonic cell sheet in *V. carteri *is achieved by cells at the bend points, which undergo a distinct transition in cell shape [20,22-24,26-29]. Moreover, concerted movements of cells with respect to the CB system are crucial for the process of inversion in *V. carteri *[20,22-24,26-29].

Most previous research on inversion was performed in *V. carteri *[20-25]; however, this process also occurs, but has been studied in much less detail, in other species of the genus *Volvox *such as *V. tertius *[30-32], *V. obversus *[33,34], *V. aureus *[35,36], *V. globator *[37,38], *V. rousseletii *[38-40] and *V. capensis *[38]. Inversion also exists in more basal volvocine relatives of *Volvox *[25,41], such as *Pleodorina californica *[42], *Eudorina elegans *[43-45], *E. indica *[46], *Pandorina morum *[47], *Platydorina caudata *[48] and *Gonium pectorale *[44,49,50], even if the process is less distinct in these species.

Inversion is the solution to the awkward inside-out situation of the embryo in multicellular volvocine algae. After completion of embryonic cleavage divisions, the embryo contains all of the cells that will be present in the adult alga, but the flagellar ends of all of the cells point toward the interior, rather than the exterior, where they need to be to function during locomotion. Moreover, in more advanced species, which already exhibit large reproductive cells before inversion (for example, *V. carteri*), the reproductive cells point outward. This inside-out orientation is corrected as the embryo turns itself right-side out during inversion.

**Figure 1:**
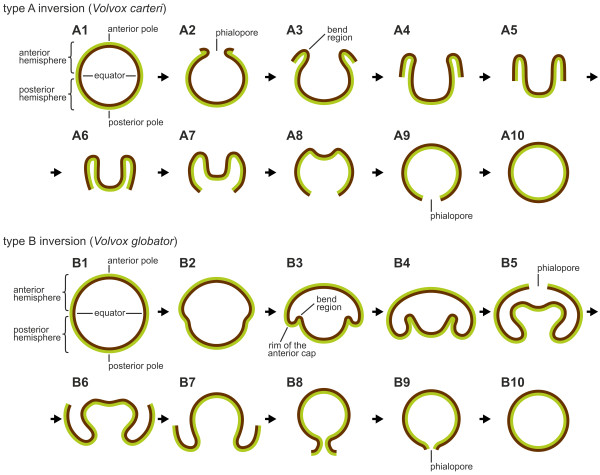
**Stylized sequences of the different inversion processes in Volvocaceae: type A and type B**. Schematic representation of midsagittal cross sections of embryos showing inversion of type A and type B inverters. (**A**) Type A inversion in *Volvox carteri*. (**A1**) Before the initiation of inversion. (**A2**) Inversion begins with the appearance of a swastika-shaped opening, the phialopore, at the anterior pole. (**A2 **to **A8**) The cell monolayer curls outward and backward over the simultaneously contracting posterior hemisphere and the phialopore widens; the posterior hemisphere moves toward the phialopore; the region of maximum cell-sheet curvature, the bend region, moves progressively to the posterior pole. (**A9**,**A10**) Inversion ends with the closure of the phialopore. (**B**) Type B inversion in *Volvox globator*. (**B1**) Before the initiation of inversion. (**B2**) Inversion begins with the contraction of the posterior hemisphere. (**B3**) Contraction proceeds and accompanies movement of the posterior hemisphere into the anterior hemisphere; this invagination of the cell sheet generates a ring of maximum curvature between the two hemispheres, the bend region. (**B4**,**B5**) The posterior hemisphere lies completely within the anterior hemisphere but moves further still in direction of the anterior pole; a circular opening, the phialopore, appears at the anterior pole and widens. (**B5 **to **B7**) The widening of the phialopore proceeds and the anterior hemisphere moves over the already inverted posterior hemisphere. (**B7**,**B8**) The last third of the anterior hemisphere moves over the already inverted cell monolayer. (**B9**,**B10**) Inversion ends with the closure of the phialopore. In both species, the side of the cell layer that is outside in the adult configuration and from which the flagella will emerge is presented in brown, and the side of the cell layer that is inside in the adult configuration and where the reproductive cells are located, is presented in green.

The process of inversion was discovered in *V. carteri f. weismannia *approximately 100 years ago, but it was initially misinterpreted as a pathological phenomenon [51]. The correct interpretation of inversion was provided by Kuschakewitsch approximately a decade later [52,53]. Subsequent work revealed that inversion in *Volvox *involves two cellular events: dramatic changes in cell shapes and coordinated movements of cells relative to the CBs [20-24,26-29,31,54-57]. However, there are two fundamentally different sequences through which embryos of the genus *Volvox *turn right-side out: type A and type B (Figure 1) [25,58]. In type A inversion, which occurs in *V. carteri *and species such as *V. obversus*, *V. africanus*, *V. gigas *and *V. tertius*, an opening, called the phialopore, appears at the anterior pole; the anterior hemisphere inverts through the phialopore; the posterior hemisphere then inverts; and finally, the phialopore closes (Figure 1A) [22,32-34,54]. (It should be noted that by convention, the anterior pole of a pre-inversion embryo becomes the posterior pole of the inverted embryo [22,25].) In type B inversion (Figure 1B), as observed in species such as *V. globator*, *V. capensis*, *V. dissipatrix*, *V. rousseletii *and *V. barberi*, the posterior hemisphere inverts before the phialopore opens, followed by inversion of the anterior hemisphere; finally, the phialopore closes [26,38-40]. Thus, in type B inversion, the opening of the phialopore does not indicate the beginning of inversion, and the hemispheres invert in reverse order compared to type A inversion.

In terms of evolution, inversion must have arisen quite recently because phylogenetic analyses indicate that multicellularity evolved in volvocine green algae 'only' approximately 200 million years ago [59], which is much more recently than in any other group, and inversion must have evolved in parallel with the transition to multicellularity or even later. Phylogenetic analyses have also indicated a polyphyletic origin of the genus *Volvox *[22,60-66] (see Additional File 1) suggesting that it is not a true genus. A few species of this genus, including *V. globator*, form a small but robust monophyletic group that is referred to as the section *Volvox *[58,62,64,67,68]. All *Volvox *species within the section *Volvox *exhibit type B inversion (see Additional File 1). The other species of the genus *Volvox *and the genera *Eudorina *and *Pleodorina *constitute another, much larger, monophyletic group, the *Eudorina *group [58,69]. The *Volvox *species within the *Eudorina *group include both type A and type B inverters (see Additional File 1).

The type A inversion of *V. carteri *has been analyzed previously in great detail (Figure 1A) [20,23-27,29,70-74]. Inversion in *V. carteri *begins about 1 h after the end of embryonic cleavages with the appearance of a swastika-shaped phialopore at the anterior pole of the embryo (Figure 1A1, A2). At this opening, the four lips of the cell monolayer curl outward and backward over the simultaneously contracting posterior hemisphere (Figure 1A2, A3, A4, A5, A6, A7, A8). During inversion, the circular region of maximum cell-sheet curvature moves progressively along the anterior-posterior axis. Finally, the phialopore closes, and the embryo regains its spherical phenotype (Figure 1A9, A10). The flagellar ends of the somatic cells now point outward, and the reproductive cells (gonidia) point inward. The entire inversion process lasts approximately 45 min [23,25]. Analyses of the cytological changes that occur during this epithelial folding using light microscopy (LM), transmission electron microscopy (TEM) and scanning electron microscopy (SEM) have led to the development of a model that describes inversion in *V. carteri *based on cell shape changes and active movements of cells relative to the CBs connecting them [20,21,27,28]. At the beginning of inversion in a *V. carteri *embryo, all cells are spindle shaped and are joined at their widest points by CBs [27]. The cells surrounding the phialopore first develop long, microtubule-reinforced stalks. In *V. carteri*, a motor protein, the kinesin InvA, is thought to generate the force that drives the movement of the cells relative to the CBs until they are linked at their narrowest, outermost tips [24,25,27]. These combined actions cause the phialopore lips in *V. carteri *to bend outward. All cells gradually undergo these changes in cell shape, and a ring of maximum curvature, known as the bend region, moves from the anterior to the posterior pole until inversion is completed (Figure 1A). After passing the region of maximum curvature, the cells, which are linked by their inner ends, develop a columnar shape.

In contrast to the type A inversion of *V. carteri*, type B inversion has only been studied by LM and, therefore, only basic information about the sequence and mechanism of inversion in type B inverters is available [25,26,36-38,54,75]. Although the subcellular mechanisms underlying inversion might be similar in type A and type B inverters, there must be significant differences between the two types of inversion to explain the different inversion sequences observed.

Therefore, in the present study, we provide a detailed description of the cellular mechanism of type B embryonic inversion in *Volvox globator*, compare type A inversion (as previously studied in *V. carteri*) and type B inversion (as studied here) in terms of the key similarities and differences in the cellular events that are involved, deal with the pathways by which these two types of inversion may have evolved and discuss a hypothesis that integrates our results and biomechanical implications about the involved tensile and compressive forces into a global mechanistic scenario that predicts epithelial folding in *V. globator*.

## Results

**Figure 2:**
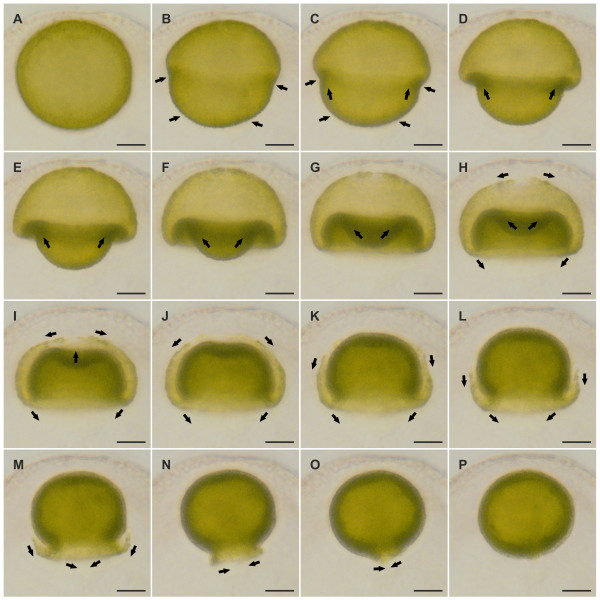
**Sequence of light micrographs of an inverting *V. globator *embryo *in situ *and *in vivo***. Time-lapse sequence of a single living embryo inside its parental spheroid. The embryo was captured in side view at 3.5-min intervals throughout inversion. (**A**) The embryo 3.5 min before the initiation of inversion. (**B**) Inversion begins with the contraction of the posterior hemisphere. (**C **to **F**) 3.5 min, 7 min, 10.5 min and 14 min after the initiation of inversion; contraction proceeds and accompanies movement of the posterior hemisphere into the anterior hemisphere; invagination of the cell sheet generates a ring of maximum curvature between the two hemispheres. (**G **to **K**) 17.5 min, 21 min, 24.5 min, 28 min and 31.5 min after initiation of inversion; the posterior hemisphere lies completely within the anterior hemisphere but moves further still toward the anterior pole; the phialopore appears at the anterior pole and widens continuously. (**H **to **L**) 21 min, 24.5 min, 28 min, 31.5 min and 35 min after the initiation of inversion; the widening of the phialopore proceeds and the anterior hemisphere moves over the already inverted posterior hemisphere. (**L **to **O**) 35 min, 38.5 min, 42 min and 45.5 min after the initiation of inversion; the last third of the anterior hemisphere moves over the already inverted cell monolayer. (**O**,**P**) 45.5 min and 49 min after the initiation of inversion; inversion ends with the closure of the phialopore. Directions of cell layer movements are indicated by black arrows. Scale bars: 20 μm.

**Figure 3:**
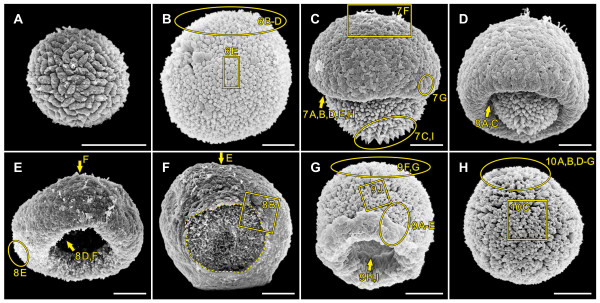
**Scanning electron micrographs of *V. globator *embryos in successive stages of inversion**. Embryos were removed from their mother spheroids and their embryonic vesicle and analyzed by scanning electron microscopy. (**A**) An early embryo during cell division (400-cell stage) several hours before the initiation of inversion. (**B**) A fully cleaved embryo 5 min to 10 min before the initiation of inversion; the embryo contains all of the cells that will be present in the adult (approximately 3,000 cells); details are shown in Figure 6B, C, D, E. (**C**) An embryo 5 min to 10 min after the initiation of embryonic inversion; details are shown in Figure 7. (**D**) An embryo 10 min to 15 min after the initiation of inversion; details are shown in Figure 8A, C. (**E**) An embryo 20 min to 25 min after the initiation of inversion, as observed from a slanted side view; details are shown in Figure 8D, E, F. (**F**) Embryo 20 min to 25 min after the initiation of inversion; view of the anterior hemisphere of the same embryo as in E; a broken line indicates the phialopore; details are shown in Figure 8B. (**G**) Embryo 35 min to 40 min after the initiation of inversion; details are shown in Figure 9. (**H**) Embryo 5 min to 10 min after the end of inversion (that is, 55 min to 60 min after the beginning of inversion); details are shown in Figure 10A, B, C, D, E, F, G. Details shown at higher magnification in the figures below are indicated by rectangles; details shown in sections or from a different angle are indicated by ovals; arrows indicate the viewing direction for regions that are shown in the referenced figures but are hidden in the given figure. Scale bars: 20 μm.

**Figure 4:**
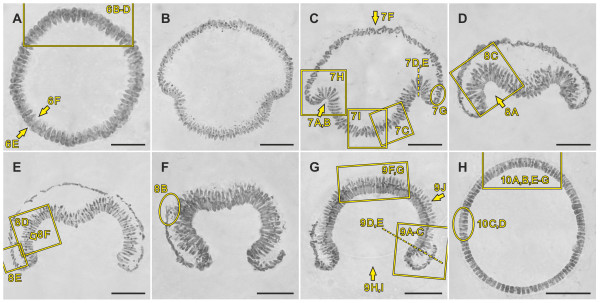
**Midsagittal sections of *V. globator *embryos in successive stages of inversion**. Stained sections were analyzed by light microscopy. (**A**) An embryo 5 min to 10 min before the initiation of inversion; details are shown in Figure 6B, C, D, E, F. (**B**) An embryo 3 min to 5 min after the initiation of inversion. (**C**) An embryo 5 min to 10 min after the initiation of inversion; details are shown in Figure 7. (**D**) An embryo 10 min to 15 min after the initiation of inversion; details are shown in Figure 8A, C. (**E**) An embryo 20 min to 25 min after the initiation of inversion; details are shown in Figure 8D, E, F. (**F**) An embryo 30 min to 35 min after the initiation of inversion; details are shown in Figure 8B. (**G**) An embryo 35 min to 40 min after the initiation of inversion; details are shown in Figure 9. (**H**) An embryo 5 min to 10 min after the end of inversion (that is, 55 min to 60 min after initiation of inversion); details are shown in Figure 10A, B, C, D, E, F, G. Rectangles, ovals and arrows indicate details shown in other figures as described in the legend to Figure 3; a broken line refers to a detailed view that is perpendicular to the image plane. Scale bars: 20 μm.

**Figure 5:**
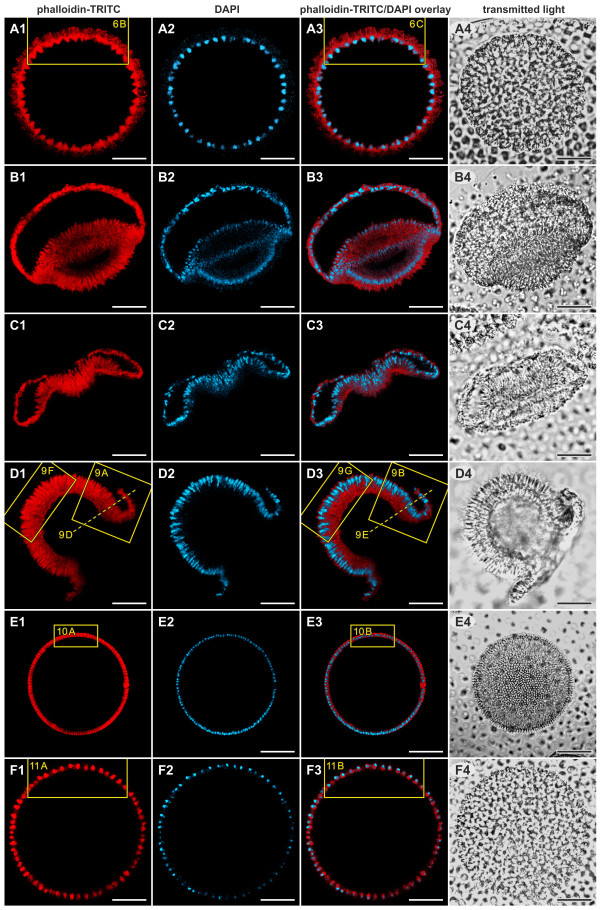
***In situ *localization of F-actin and nuclei in optical sections of successive stages of inversion**. cLSM and transmitted light images of intact embryos. F-actin was visualized with phalloidin-TRITC (red, first column), and DNA was stained with DAPI to visualize nuclei (blue, second column). The third column shows phalloidin-TRITC/DAPI overlays, and the fourth column shows transmitted light brightfield micrographs. (**A1 **to **A4**) An embryo 5 min to 10 min before the initiation of inversion (A1,2,3, midsagittal section); details are shown in Figure 6B, C. (**B1 **to **B4**) An embryo 5 min to 10 min after the initiation of inversion (B1,2,3, oblique lateral section); the embryo was somewhat compressed by the weight of the coverslip. (**C1 **to **C4**) An embryo 20 min to 25 min after the initiation of inversion (C1,2,3, midsagittal section). (**D1 **to **D4**) An embryo 35 min to 40 min after the initiation of inversion (D1,2,3, midsagittal section); details are shown in Figure 9A, B, D, E, F, G. (**E1 **to **E4**) An embryo 5 min to 10 min after the end of inversion, that is, 55 min to 60 min after the initiation of inversion (E1,2,3, midsagittal section); details are shown in Figure 10A, B. (**F1 **to **F4**) An embryo approximately 1 h after the end of inversion, that is, approximately 2 h after the initiation of inversion (F1,2,3, midsagittal section); details are shown in Figure 11A, B. Details that are shown at higher magnification in figures below are indicated by rectangles; broken lines refer to detailed views that are perpendicular to the image plane. Scale bars: 20 μm. cLSM: confocal laser scanning microscopy; DAPI: 4',6-diamidino-2-phenylindole dihydrochloride; F-actin: filamentous actin; TRITC: tetramethylrhodamine B isothiocyanate.

Epithelial folding of the typical type B inverter *V. globator *(Figure 1) was investigated in detail, focusing on major movement patterns of the cellular monolayer, region and stage-specific changes in cell shape and changes in the localization of CBs. To describe the major aspects of inversion separately but in ontogenetic order of appearance, we go through the inversion process several times by calling attention to the relevant panels of Figures 2, 3, 4 and 5; each of these figures shows an image series of inversion in ontogenetic order made with four different techniques: LM time-lapse *in vivo *imaging (Figure 2), SEM (Figure 3), LM of mechanical, midsagittal sections (Figure 4) and optical sections by confocal laser scanning microscopy (cLSM) (Figure 5).

### Major movement patterns of the cellular monolayer

#### Pre-inversion stage

**Figure 6:**
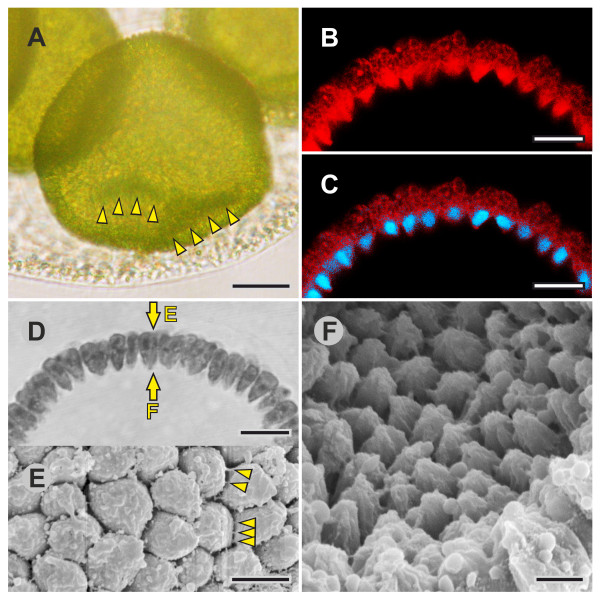
**Details of embryos 5 min to 10 min before the initiation of inversion (pre-inversion stage)**. cLSM, SEM and LM images of intact or fragmented embryos, and an LM image of a physical cross-section. (**A**) LM side view of a juvenile inside the intact parental spheroid. Arrowheads point to minor dents that quickly appear and disappear immediately before the initiation of inversion. (**B**) Midsagittal cLSM optical cross-section of an embryo showing the localization of F-actin (red) using phalloidin-TRITC (see Figure 5A1 for an overview). (**C**) Midsagittal cLSM optical cross-section of an embryo showing the localization of both F-actin (red) and nuclei (blue) with a phalloidin-TRITC/DAPI overlay (see Figure 5A3 for an overview). (**D**) LM image of a physical midsagittal cross-section. Arrows indicate the viewing direction in (E) and (F) (see Figure 4A for an overview). (**E**) SEM view of the cell monolayer observed from outside an embryo (see D and Figure 3B for an overview); arrowheads point to some of the numerous CBs. (**F**) SEM view of the cell monolayer observed from inside a fragmented embryo (see D for an overview). Scale bars: (A) 20 μm; (B, C, D) 10 μm; (E, F) 3 μm. CB: cytoplasmic bridge; cLSM: confocal laser scanning microscopy; DAPI: 4',6-diamidino-2-phenylindole dihydrochloride; F-actin: filamentous actin; LM: light microscopy; SEM: scanning electron microscopy; TRITC: tetramethylrhodamine B isothiocyanate.

At the stage immediately (5 min to 10 min) before the beginning of inversion, a *V. globator *embryo is fully cleaved. The embryo completed 11 to 13 rounds of cell division and has 2,000 to 6,000 cells arranged in a spherical cellular monolayer with a diameter of approximately 80 μm (70 μm to 90 μm) (Figures 1B1, 2A, 3B, 4A and 5A). At this time, the spheroidal embryo exhibits sporadic movements of the cell monolayer, and several dents quickly appear and disappear (Figure 6A). This phenomenon was also observed in *V. carteri*, *V. aureus *and *V. rousseletii *embryos and is known as 'denting' [20,26,39]. Denting ends once the process of inversion starts.

#### Early inversion stage

**Figure 7:**
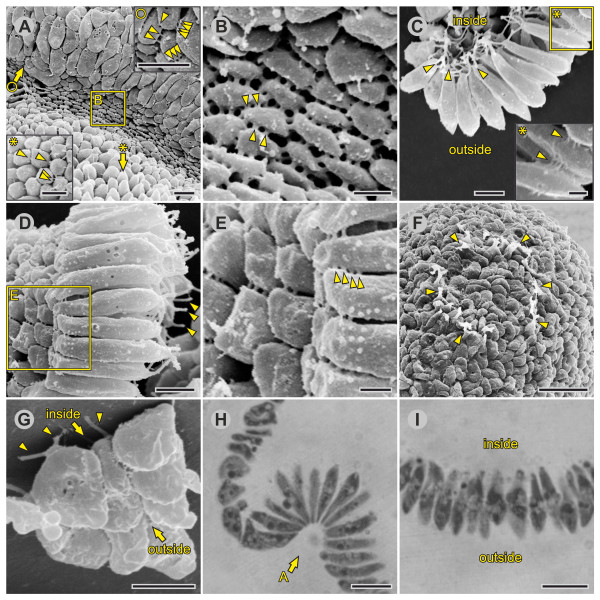
**Detailed characteristics of embryos 5 min to 10 min after the initiation of inversion (early inversion stage)**. SEM images of isolated intact or fragmented embryos and LM images of physical midsagittal cross-sections (see Figures 3C and 4C for an overview). (**A**) SEM view of the bend region; the anterior hemisphere is in the upper part of the image; arrow with asterisk: the viewing direction of the cells from the posterior hemisphere shown in the inset in the lower left corner; arrow with circle: the area shown in the inset in the upper right corner; arrowheads: CBs; rectangle: detail shown in (B). (**B**) SEM view of cells in the bend region; arrowheads: CBs. (**C**) SEM side view of cells of the posterior hemisphere (fragmented embryo); arrowheads: outgrowing flagella; rectangle: detail shown in the inset; arrowheads in the inset: CBs. (**D**) Slanted SEM side view of cells of the bend region (fragmented embryo); arrowheads: outgrowing flagella; rectangle: detail shown in (E). (**E**) Slanted SEM side view of the chloroplast ends of the cells shown in (D); arrowheads: CBs. (**F**) SEM view of the anterior pole of the embryo; arrowheads: ring of broken CBs that connected the embryo with the somatic cells of its parent spheroid. (**G**) Slanted SEM top view of cells of the anterior hemisphere (fragmented embryo); arrowheads: outgrowing flagella. (**H**, **I**) LM images of a physical midsagittal cross-section of the bend region and the posterior hemisphere, respectively. Arrow in (H): the viewing direction of the cells shown in (A); however, the stage in (H) is somewhat earlier than the stage in (A). (C, G and I) The inside and outside of the embryo are indicated. Scale bars: (A, C, D, G and insets in A) 3 μm; (B, E and inset in C) 1 μm; (F) 10 μm; (H, I) 5 μm. CB: cytoplasmic bridge; LM: light microscopy; SEM: scanning electron microscopy.

The inversion process begins with the contraction of the posterior hemisphere of the embryo (Figures 1B2, 2B and 4B). During the first approximately 10 min of inversion, contraction proceeds and accompanies movement of the posterior hemisphere in the direction of the anterior pole; thus, the posterior hemisphere moves into the anterior hemisphere (Figures 1B2, B3, 2B, C, D, E, F, 3C, 4B, C and 5B). This ring-shaped invagination of the cell sheet generates the bend region (Figures 1B3, 2B, C, D, 3C, 4B, C and 7A). In midsagittal cross-sections of embryos at this stage, the bend region forms more or less a half circle with a radius of approximately 2 μm (Figure 7H).

#### Mid-inversion stage

**Figure 8:**
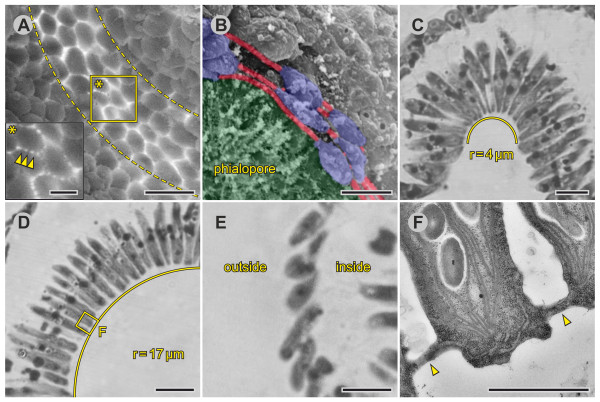
**Details of embryos 10 min to 35 min after the initiation of inversion (mid-inversion stage)**. SEM images of isolated embryos and LM and TEM images of physical midsagittal cross-sections (see Figures 3D, E, F and 4D, E, F for an overview). (**A**) SEM view of the bend region; two broken lines encompass the region with maximum curvature; a rectangle indicates an image detail shown in the inset; and arrowheads point to some of the numerous CBs. (**B**) SEM view of the opening of the phialopore; the anterior hemisphere is in the upper right corner, and the already inverted posterior hemisphere is in the lower left corner (highlighted in green); passively stretched cells bordering the phialopore are colored in blue; and CBs are colored in red. (**C**, **D**) LM images of physical midsagittal cross-sections of the bend region. The developmental stage in (D) is approximately 10 min later than the stage in (C); during this period, the radius of the curvature in the bend region increases significantly as indicated; and a rectangle indicates an image detail shown in (F). (**E**) LM image of a physical midsagittal cross-section with cells of the anterior hemisphere; the inside and outside of the embryo are indicated. (**F**) TEM image of a physical midsagittal cross-section of the bend region showing the position of CBs (arrowheads) (see D for an overview). Scale bars: (A, E) 3 μm; (B, C, D) 5 μm; (F and inset in A) 1 μm. CB: cytoplasmic bridge; LM: light microscopy; SEM: scanning electron microscopy; TEM: transmission electron microscopy.

The mid-inversion stage spans from approximately 10 min to approximately 35 min after the beginning of inversion. Approximately 15 min to 20 min after the initiation of inversion, the posterior hemisphere lies completely within the anterior hemisphere and moves further yet toward the anterior pole (Figures 1B4, 2G, H, I, J, K, 3D, E, 4D, E, F and 5C). During this movement of the posterior hemisphere, the radius of the curvature in the bend region increases considerably from 3 μm to 5 μm up to 15 μm to 20 μm within approximately 10 min (Figure 8C, D). Approximately 20 min to 25 min after the beginning of inversion, a circular opening, the phialopore, appears at the anterior pole (Figures 1B5, 2G, H and 3F). The phialopore widens continuously while the anterior hemisphere moves over the already almost completely inverted posterior hemisphere (Figures 1B5, B6, 2H, I, J, K, L, 3F, 5C and 8B). The inner diameter of the opening increases until it is somewhat greater than the outer diameter of the inverted posterior hemisphere (Figures 1B6 and 2K, L).

#### Late inversion stage

During the late stage of inversion, 35 min to 45 min after its onset, the last third of the anterior hemisphere moves over the already inverted cell monolayer (Figures 1B7, B8, B9, 2L, M, N, O, 3G, 4G, 5D and 9A, B, C). During this last part of inversion, the diameter of the opening decreases continuously (Figures 1B9 and 2L, M, N, O). Approximately 50 min after it started, inversion ends with the closure of the phialopore (Figures 1B10 and 2O, P).

**Figure 9:**
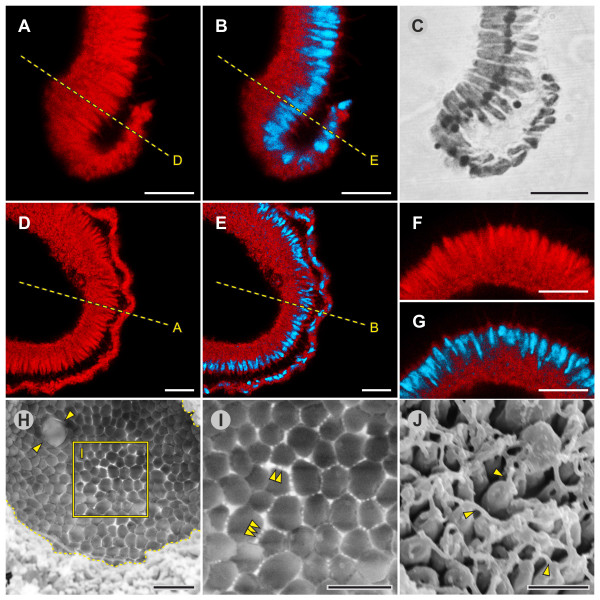
**Details of embryos 35 min to 40 min after the initiation of inversion (late-inversion stage)**. cLSM and SEM images of intact embryos and an LM image of a physical cross-section. (**A**, **F**) Midsagittal cLSM optical cross-section of an embryo showing the localization of F-actin (red) using phalloidin-TRITC (see Figure 5D1 for an overview). (**B**, **G**) Midsagittal cLSM optical cross-section of an embryo showing the localization of both F-actin (red) and nuclei (blue) with a phalloidin-TRITC/DAPI overlay (see Figure 5D3 for an overview). (**C**) LM image of a physical midsagittal cross-section (see Figure 4G for an overview). (**D**, **E**) cLSM optical cross-sections perpendicular to the midsagittal cross-sections showing the localization of (D) F-actin (red) alone or (E) both F-actin (red) and the nuclei (blue). (**H**) SEM view of a mostly inverted embryo (see Figures 3G and 4G for an overview); a broken line indicates the edge of the opening, and a rectangle highlights an image detail shown in (I); and arrowheads point to a reproductive cell (gonidium). (**I**) Detailed SEM view of the cell monolayer from inside the spheroid (see H for an overview); arrowheads point to some of the numerous CBs. (**J**) Detailed SEM view of the cell monolayer from outside the spheroid (see Figures 3G and 4G for an overview); arrowheads point to some of the outgrowing flagella. (A, B, D, E) Broken lines refer to views that are perpendicular to the image plane. Scale bars: (A, B, C, D, E, F, G) 10 μm; (H) 5 μm; (I, J) 3 μm. CB: cytoplasmic bridge; cLSM: confocal laser scanning microscopy; DAPI: 4',6-diamidino-2-phenylindole dihydrochloride; F-actin: filamentous actin; LM: light microscopy; SEM: scanning electron microscopy; TRITC: tetramethylrhodamine B isothiocyanate.

#### Early and late post-inversion stage

**Figure 10:**
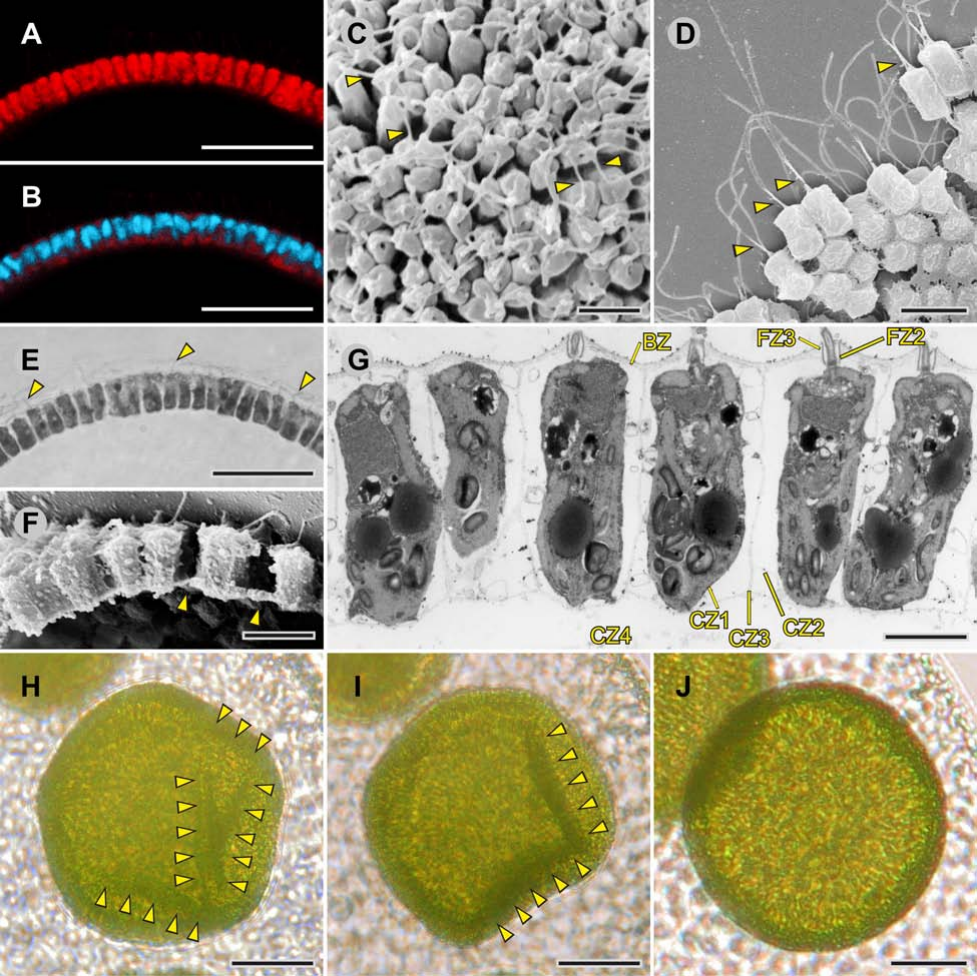
**Details of juveniles 5 min to 10 min after the completion of inversion (early post-inversion stage)**. cLSM, SEM and LM images of intact or fragmented juveniles and LM and TEM images of physical cross-sections. (**A**) Midsagittal cLSM optical cross-section of a juvenile showing the localization of F-actin (red) using phalloidin-TRITC (see Figure 5E1 for an overview). (**B**) Midsagittal cLSM optical cross-section of a juvenile showing the localization of both F-actin (red) and nuclei (blue) with a phalloidin-TRITC/DAPI overlay (see Figure 5E3 for an overview). (**C**) SEM view of the cell monolayer from outside the spheroid (see Figures 3H and 4H for an overview); arrowheads point to some of the flagella. (**D**) Slanted SEM side view of cells (fragmented juvenile); arrowheads point to the flagella. (**E**) LM image of a physical midsagittal cross-section (see Figures 3H and 4H for an overview); arrowheads point to some of the flagella. (**F**) SEM side view of cells of the cell monolayer (fragmented juvenile); arrowheads point to the CBs (see Figures 3H and 4H for an overview). (**G**) TEM image of a physical midsagittal cross-section of the cell monolayer showing some of the zones of the ECM (see Figures 3H and 4H for an overview); flagellar zone (FZ), boundary zone (BZ), cellular zone (CZ) and ECM zones and subzones are described in [77] and [78]. (**H **to **J**) LM side view of a juvenile inside the intact parental spheroid. (H, I) Arrowheads point to minor dents that quickly appear and disappear immediately after inversion. (J) After the short period of denting, the embryos remain spherical. Scale bars: (A, B, E) 10 μm; (C) 3 μm; (D, F) 5 μm; (G) 1 μm; (H, I, J) 20 μm. BZ: boundary zone; CB: cytoplasmic bridge; CZ, cellular zone; cLSM: confocal laser scanning microscopy; DAPI: 4',6-diamidino-2-phenylindole dihydrochloride; ECM: extracellular matrix; F-actin: filamentous actin; FZ: flagellar zone; LM: light microscopy; SEM: scanning electron microscopy; TEM: transmission electron microscopy; TRITC: tetramethylrhodamine B isothiocyanate.

Immediately after inversion, the embryo has a diameter of only approximately 60 μm (50 μm to 70 μm) (Figures 2P, 3H, 4H and 5E), which is approximately 20 μm less than at the initiation of inversion (Figures 2A, 3B, 4A and 5A). Not only before (see above) but even shortly after the completion of inversion, we observed sporadic movements of the cell monolayer causing dents that quickly appear and disappear (Figure 10H, I). To our knowledge, this denting after inversion has never been observed in any other *Volvox *species. Approximately 1 h after the completion of inversion, denting is no longer observed (Figure 10J), and the juveniles remain spheroidal.

### Region and stage-specific changes in cell shape

#### Pre-inversion stage

During cleavage, all cells are roughly spherical or, particularly immediately before each cleavage division, somewhat elongated (Figure 3A). The cells exhibit a length of 4.5 μm to 6.5 μm and a width of 2.5 μm to 5 μm. Between the end of cleavage and the beginning of inversion, all cells within the spherical cellular monolayer develop into what we refer to as teardrop-shaped cells (Figures 3B, 4A, 5A1, A3, and 6B, C, D, E, F). Each of these approximately radially symmetrical cells is cone-shaped at the cell end that faces the interior (Figure 6B, D, F); we refer to the cone-shaped (apical) end of the cell as the flagellar end because the flagella will develop on this side of the cell. The opposite end of the teardrop-shaped cells, facing the exterior, shows a roughly hexagonal cross-section (Figure 6E); we call this end of the cell the chloroplast end because the single chloroplast largely localizes on this side of the cell. Teardrop-shaped cells have a length of 8 μm to 10 μm and a maximal width of 3 μm to 5 μm.

For all optical sections of embryos, we used phalloidin-tetramethylrhodamine B isothiocyanate (TRITC) as a cytoplasmic stain because it stains filamentous actin (F-actin) in embryonic cells [72], and F-actin was more or less uniformly distributed in the cytoplasm (both in this and in a previous study [72], the resolution of the cLSM was insufficient to see individual actin filaments). However, there was one exception: in teardrop-shaped cells, F-actin was mainly localized at the flagellar (apical) ends of the cells (Figure 6B, C).

#### Early inversion stage

At the beginning of inversion, the cells of the anterior hemisphere remain teardrop shaped (Figure 4B, C), but the cells of the posterior hemisphere become elongated along their chloroplast-end-to-flagellar-end axis. The latter cells attain a length of 10 μm to 12 μm with more or less pointed ends on both sides; therefore, these cells are referred to as spindle-shaped cells (Figures 4B, C, 5B1, B3 and 7C, I). At the cell's equator, which is the broadest point, the spindle-shaped cells have a diameter of 2 μm to 3 μm and exhibit a hexagonal cross-section (Figure 7C and inset in 7C).

The conversion of teardrop-shaped cells into spindle-shaped cells in the posterior hemisphere occurs in conjunction with the formation of the bend region somewhat below the equator of the embryo (Figures 1B1, B2, 2A, B, 3C, 4B, C and 7H). In the emerging bend region, the shapes of the just developed spindle cells changes again and increasingly reflects the maximum curvature of the cell sheet in this area. The elongated cells of this region stay pointed and radially symmetrical only at their flagellar ends, whereas they become increasingly wedge-shaped at their chloroplast ends; we refer to these cells as paddle-shaped cells. In a cross-section of the embryo, the cells of the bend region are arranged in the form of a spread fan (Figures 4C and 7H), exhibit a length of 9 μm to 10 μm and are approximately 2 μm to 3 μm in diameter at the broadest point (Figure 7D). At the outermost chloroplast ends, the major axis of the cell's cross-section is 1.5 μm to 2 μm, while the minor axis is only approximately 0.5 μm (middle part of Figure 7A, B); that is, there is a difference in length by a factor of three to four between the two axes. The bases of the cells at the chloroplast ends frequently appear as elongated hexagons. The major axis of these elongated hexagons at the outermost chloroplast ends of paddle-shaped cells is oriented along a circular line described by the ring-shaped invagination of the posterior hemisphere; this circular line shows only minor curvature due to its relatively large radius of approximately 30 μm. The minor axis of the outermost chloroplast end of paddle-shaped cells is perpendicular to the circular line around the embryo and is oriented along a line that more or less forms a half circle in the bend region when a midsagittal cross-section of an embryo is observed (Figures 4C and 7H).

While invagination of the posterior hemisphere proceeds and the transition from spindle to paddle shapes progresses from the equator toward the posterior pole, the teardrop-shaped cells of the anterior hemisphere become increasingly elongated flat cells that partially overlap each other. The conversion from teardrop-shaped to elongated progresses from the rim of the anterior cap of the embryo toward its anterior pole. We refer to the elongated flat cells as disc shaped (Figures 4B, C and 7A (upper right part of the image) and 7G). However, these cells are not uniformly shaped, and quite a few are buckled. Disc-shaped cells are elongated in the anterior-posterior direction of the embryo and frequently have a length of 4 μm to 6 μm, a width of 2 μm to 3.5 μm and a height of 0.8 μm to 2 μm.

#### Mid-inversion stage

At the beginning of the mid-inversion stage, the cells in the bend region still have the appearance of paddles that are flattened at one end (the chloroplast end) and are arranged in the form of a spread fan (Figures 4D and 8A, C) but at this stage, these cells have a length of 11 μm to 12 μm and, thus, are longer than the corresponding cells in the early inversion stage. The bases of the paddle-shaped cells at the chloroplast ends still have the appearance of elongated hexagons (Figure 8A).

In the mid-inversion stage, the transition from the remaining spindle to paddle cells seems to progress from the equator of the embryo toward its posterior pole and, as mentioned above, the radius of the curvature in the bend region increases considerably. The greater that the radius of the curvature in the bend region is, the thicker the chloroplast ends are on their narrow side (Figures 4D, E and 8C, D). With a further increase of this radius, the paddle-shaped cells change into cells that are radially symmetrical throughout their length (Figures 4E, F and 8D); these cells still have pointed flagellar ends but the bases of the cells at the flattened chloroplast ends appear as elongated hexagons (Figures 4E, F and 8A, D, F), and we refer to these cells as pencil shaped. Pencil-shaped cells have a length of 11.5 μm to 12.5 μm and a diameter of 1.5 μm to 2 μm (at the chloroplast end).

During most of the mid-inversion stage, cells close to the not yet inverted posterior pole are still spindle-shaped (Figure 4D, E). When these cells have their turn in inversion, the radius of the curvature in the bend region reaches its maximum. These spindle-shaped cells do not seem to become paddle shaped but directly change into the pencil shape (Figure 4D, E, F).

Before inversion of the posterior hemisphere is complete, disc-shaped cells at the rim of the anterior cap become increasingly pencil shaped (also without passing through the paddle stage) and, at the same time, seem to move over the rim of the anterior cap toward the posterior pole (Figures 3E, F, G, 4C, D, E and 5C1, C3). This process appears to be the initiation of inversion of the anterior hemisphere. Shortly after the beginning of the change from disc to pencil shape at the rim of the anterior cap, the phialopore appears at the anterior pole and the opening widens continuously. The transition from disc to pencil shape seems to progress toward the phialopore. Disc-shaped cells around the phialopore alone become increasingly elongated along a circular line described by the edge of the opening (Figures 3F and 8B). Cells in the not yet inverted part of the anterior hemisphere are still disc shaped (Figures 4E, F, 5C1, C3 and 8E).

#### Late inversion stage

During this last stage of inversion, the remaining disc-shaped cells of the not yet inverted portion of the anterior hemisphere become pencil-shaped (Figures 3G, 4G, 5D1, D3 and 9A, C, D, F) and simultaneously seem to move over the rim of the remaining portion of the anterior cap. In the late inversion stage, pencil-shaped cells have a length of 11.5 μm to 12.5 μm, are 1.5 μm to 2 μm in diameter, have a more or less hexagonal base at their chloroplast ends (Figure 9H, I, J) and show pointed flagellar ends (Figure 9J). Once all cells changed into pencil shape, the phialopore closes and inversion is completed.

#### Early and late post-inversion stage

Right after inversion is complete, the cells of the embryo change their shape again, but this time all cells do it concertedly. The pencil-shaped cells become shorter and end up at a length of 4.5 μm to 6.5 μm and as a result the diameter increases to 2 μm to 4 μm (Figures 3H, 4H, 5E1, E3 and 10A, C, D, E, F, G). We refer to these cells as column shaped. The column-shaped cells present a hexagonal cross-section along their entire length, except for at the slightly rounded flagellar ends, while the chloroplast ends are flattened (Figure 10C, D).

Approximately 1 h after the completion of inversion, the cell shapes change for the last time: all the column-shaped cells gradually develop into what we refer to as gemstone-shaped cells (Figures 5F1, F3 and 11A). At the flagellar end, these cells exhibit shapes similar to flat, radially symmetrical cones but have more or less hexagonal bases at their chloroplast ends. The cells with the final gemstone shape have a diameter of 4.5 μm to 5 μm and a height of 5 μm to 6 μm.

**Figure 11:**
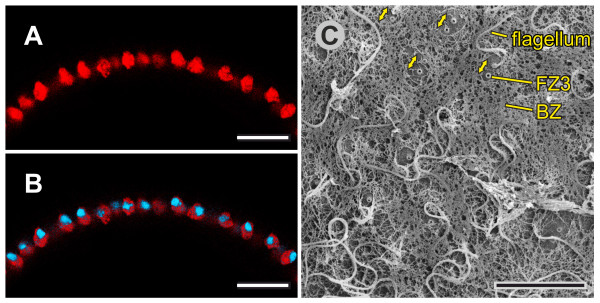
**Details of juveniles approximately 1 h after the completion of inversion (late post-inversion stage)**. cLSM and SEM images of intact or fragmented juveniles. (**A**) Midsagittal cLSM optical cross-section of a juvenile showing the localization of F-actin (red) with phalloidin-TRITC (see Figure 5F1 for an overview). (**B**) Midsagittal cLSM optical cross-section of a juvenile showing the localization of both F-actin (red) and nuclei (blue) with a phalloidin-TRITC/DAPI overlay (see Figure 5F3 for an overview). (**C**) SEM view of the cell monolayer from outside the spheroid; the conspicuous zones of the ECM are the BZ and FZ3, as described in [77] and [78]; and at regions where flagella partially broke off during preparation, double-headed arrows highlight the regular distance and orientation of flagellar pairs. Scale bars: (**A**, **B**, **C**) 10 μm. BZ: boundary zone; cLSM: confocal laser scanning microscopy; DAPI: 4',6-diamidino-2-phenylindole dihydrochloride; ECM: extracellular matrix; F-actin: filamentous actin; FZ: flagellar zone; SEM: scanning electron microscopy; TRITC: tetramethylrhodamine B isothiocyanate.

### Region- and stage-specific changes in the locations of CBs

#### Pre-inversion stage

At the pre-inversion stage, the CB system connects all teardrop-shaped cells at their chloroplast ends (Figures 6E); there are no CBs at the flagellar ends (Figure 6D, F).

#### Early inversion stage

In early inversion, the teardrop-shaped cells of the anterior hemisphere are still connected at their chloroplast ends, but the cells of the posterior hemisphere with the just developed spindle shape have their CBs in the equatorial region of the cells (Figure 7C and inset in 7C). At the same time that the bend region forms between the two hemispheres, the spindle-shaped cells in the equatorial region of the embryo seem to move concertedly relative to the CB system until the CBs localize at their chloroplast ends; this transition seems to progress from the equator of the embryo toward its posterior pole. Cells in the bend region with the just developed paddle shape exhibit CBs only at their outermost chloroplast ends (Figure 7A, B, D, E, H), which allows for the smallest possible radius of the curvature of the cell monolayer. In disc-shaped cells, which result from the conversion of teardrop-shaped cells in the anterior hemisphere, the cytoplasmic connections to other cells are found at the edges of the discs (Figure 7A (upper right part of the image) and 7G).

Despite beginning the inversion process, the *V. globator *embryo is still connected with the parent spheroid (in contrast to the *V. carteri *embryo [22]). In the *V. globator *embryo, a ring of cells at the anterior pole maintain CBs to the somatic cells of its parent (Figure 7F).

#### Mid-inversion stage

Inversion progresses during the mid-inversion stage, and the CBs localize in spindle-, paddle- and disc-shaped cells just as described for the early inversion stage. The CBs of the just developed pencil-shaped cells of the already inverted posterior hemisphere localize at their outermost chloroplast ends (Figure 8F), just as in paddle-shaped cells.

When the phialopore opens in the mid-inversion stage, the CBs between cells at the anterior pole of the embryo and somatic cells of its parent break off mechanically. While the phialopore widens continuously, the CBs around the phialopore become elongated along a circular line described by the edge of the opening (Figure 8B). These elongated CBs reach a length of up to 10 μm.

#### Late inversion and post-inversion stage

During the last part of the inversion process, the CBs localize in disc- and pencil-shaped cells (Figure 9H, I) just as described above. After inversion, CBs connect the just developed column-shaped cells (which result from the conversion of pencil-shaped cells) at the chloroplast ends (Figure 10F). All cells remain interconnected by CBs after inversion and throughout their lifetime (in contrast to cells of *V. carteri *[22]).

### Changes in shapes of nuclei, growth of flagella and biosynthesis of extracellular matrix

Before inversion, the nuclei of all teardrop-shaped cells are either spherical or somewhat conical and are localized at the flagellar ends of the cells; the nuclei have a diameter of 3 μm to 4 μm (Figures 5A2, A3 and 6C). During inversion of a *V. globator *embryo, the shapes of the nuclei undergo major changes (in contrast to the nuclei of a *V. carteri *embryo [20]), but the shapes always reflect the shapes of the corresponding cells (Figures 5B2, B3, C2, C3, D2, D3, E2, E3, 9B, E, G and 10B). Nuclei of spindle-, disc-, paddle-, pencil- and column-shaped cells are elongated along the chloroplast-end-to-flagellar-end axis and have a length of 3 μm to 5 μm and a width of 1 μm to 2.5 μm (Figures 5B2, B3, C2, C3, D2, D3, E2, E3, 9B, E, G and 10B). The nuclei of gemstone-shaped cells are spherical with a diameter of 2 μm to2.5 μm (Figures 5F2, F3 and 11B).

The outgrowth of the flagella starts around the beginning of embryonic inversion. In the early inversion stage, cells of the embryo show short flagellar stubs, approximately 2 μm in length, at their flagellar ends (Figure 7C, D, G). During inversion, the flagella of a *V. globator *embryo grow continuously (in contrast to the flagella of a *V. carteri *embryo [76]), from approximately 2 μm to around 15 μm to 17 μm at the stage immediately after inversion (Figure 10D). In adult spheroids, the flagella reach a length of 20 μm to 25 μm; thus, immediately after inversion, the flagella have reached two-thirds of their final length. However, these growing flagella have already begun to beat; therefore, the juveniles rotate (slowly) within the parent spheroid.

The synthesis of extracellular matrix (ECM) begins at approximately the end of inversion. The main zones and subzones of the ECM are detectable even in this early stage of ECM synthesis (Figure 10G) [77,78]. In particular, the characteristic ECM subzone cellular zone 3 (CZ3) becomes apparent; CZ3 consists of coherent fibrous material that creates honeycomb-like chambers at a significant distance around individual cells (Figure 10G). After inversion, the juveniles continuously grow in size by depositing increasing amounts of ECM. The rapid growth of juveniles after inversion is only affected by the secretion of ECM material, which causes each cell to move away from its neighbors (Figure 11).

### Modeling of cell shapes, CB locations and illustration of the entire inversion process

**Figure 12:**
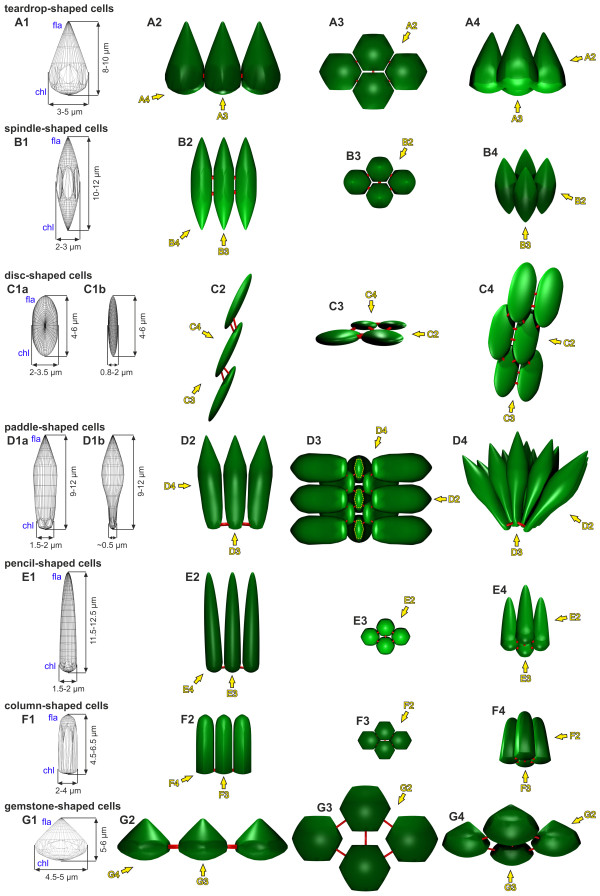
**3D representation of cell shapes and localization CBs observed before, during and after embryonic inversion**. The localization of the different cell shapes within the embryo is indicated in Figure 13.(**A1 **to **A4**) Teardrop-shaped cells: radially symmetrical cells that are cone shaped at the flagellar ends but have hexagonal cross-sections at the chloroplast ends (Figure 13A, B, C, D). (**B1 **to **B4**) Spindle-shaped cells: radially symmetrical, elongated cells with pointed ends on both sides (Figure 13B, C, D, E). (**C1 **to **C4**) Disc-shaped cells: elongated, flat cells that partially overlap each other; (**C1a**) broad and (**C1b**) narrow side of the cell (Figure 13C, D, E, F, GH). (**D1 **to **D4**) Paddle-shaped cells: elongated cells that are pointed and radially symmetrical only at the flagellar ends but wedge shaped at the chloroplast ends (Figure 13C, D); (**D1a**) broad and (**D1b**) narrow side of the cell. (**E1 **to **E4**) Pencil-shaped cells: radially symmetrical, elongated cells that are pointed at the flagellar ends and flattened at the chloroplast ends (Figure 13E, F, G, H). (**F1 **to **F4**) Column-shaped cells: radially symmetrical, elongated cells that are flattened at the chloroplast ends and rounded at the flagellar ends (Figure 13I). (**G1 **to **G4**) Gemstone-shaped cells: radially symmetrical cells that are shaped like flat cones at the flagellar ends but have hexagonal cross-sections at the chloroplast ends.(**A1 **to **G1**) Wire frame models; cell sizes and the flagellar (fla) and chloroplast (chl) ends of the cells are indicated. All other parts of the figure depict the *in vivo *arrangement of the cells (with a green surface texture) from different viewing directions. The positions of CBs are indicated by red connections between cells. (**A2 **to **G2**) Frontal side view. (**A3 **to **G3**) View of the chloroplast ends. (**A4 **to **G4**) Slanted side view. Arrows indicate the viewing directions in other parts of the figure. CB: cytoplasmic bridge.

To model cell shapes, the locations and dimensions of numerous cells on LM, cLSM, SEM and TEM images of intact and sectioned embryos were determined before, during and after inversion (see Methods). Based on these results, we developed 3D models with consensus cell shapes for the teardrop-, spindle-, disc-, paddle-, pencil-, column- and gemstone-shaped cells (Figure 12A, B, C, D, E, F, G). Moreover, we determined the locations of CBs for each cell shape, particularly in relation to the chloroplast-end-to-flagellar-end axis (Figures 12A, B, C, D, E, F, G), and we established consensus curvatures of the cell monolayer throughout all stages of inversion using LM, cLSM and SEM images.

**Figure 13:**
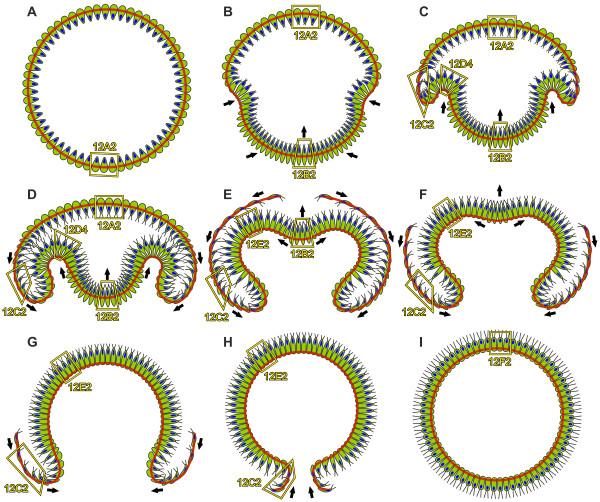
**Model of the inversion process in *V. globator***. A diagrammatic representation of inversion as deduced from the LM, SEM, TEM and cLSM analyses showing 2D midsagittal cross sections of inverting embryos. Cell content is shown in green; red lines indicate the position of the CB system; and nuclei are shown in blue. Frames show examples of the location of the cell shapes represented in 3D in Figure 12. The directions of the cell layer movements are indicated by black arrows. (**A**) Pre-inversion stage. (**B**, **C**) Early inversion stage; inversion begins with the contraction of the posterior hemisphere and the formation of the bend region. (**D **to **F**) Mid-inversion stage; the posterior hemisphere continuously moves into the anterior hemisphere and inverts at the same time; the phialopore opens at the anterior pole and widens; and the anterior hemisphere begins to move over the already inverted posterior hemisphere and inverts at the same time. (**G**, **H**) Late inversion stage; the anterior hemisphere continues to move over the already inverted cell monolayer; and inversion ends with the closure of the phialopore. (**I**) Early post-inversion stage. CB: cytoplasmic bridge; cLSM: confocal laser scanning microscopy; LM: light microscopy; SEM: scanning electron microscopy; TEM: transmission electron microscopy.

Based on all of our findings, we developed a summary model for the complete process of embryonic type B inversion in *V. globator*, which is presented in Figure 13. The model includes the localization of the observed cell shapes; the directions of cell layer movements; the relative position of the CB system; the length of flagella; and the localization and shapes of nuclei during the pre-, early, mid-, late and post-inversion stages. The summary model is discussed below.

## Discussion

### Comparison of the type A and type B inversions

When the type B inversion of *V. globator *is compared to the type A inversion of *V. carteri*, both similarities and differences are apparent (Table 1). The sequence of the major movement patterns of the cellular monolayer is quite different in both species. In *V. carteri *embryos, the phialopore opens; the anterior hemisphere inverts; the posterior hemisphere contracts; the posterior hemisphere inverts; and finally, the phialopore closes [20,22,26] (Figure 1A). In *V. globator *embryos, the posterior hemisphere contracts; the posterior hemisphere inverts; the phialopore opens; the anterior hemisphere inverts; and the phialopore closes [25,36,37] (Figures 1B and 13).

**Table 1:** Comparison of inversion in *V. globator *and *V. carteri*.

Characteristic	*V. carteri*	*V. globator*
Type of inversion	A	B

Duration of inversion	Approximately 45 min [20,22]	Approximately 50 min

Sequence of processes	The phialopore opens; the anterior hemisphere inverts; the posterior hemisphere contracts; the posterior hemisphere inverts; the phialopore closes [20,22,26]	The posterior hemisphere contracts; the posterior hemisphere inverts; the phialopore opens; the anterior hemisphere inverts; the phialopore closes [25,36,37]

Site of the initiation of inversion	Point-shaped initiation site at the anterior pole of the spheroid [22]	Ring-shaped initiation site at the equator of the spheroid

Direction of cell shape changes	From the anterior pole to the posterior pole [22]	Initially, from the equator to the posterior pole; then, from the equator to the anterior pole

Diameter of embryo	Immediately before inversion: approximately 65 μm [22] immediately after inversion: approximately 60 μm [22]	Immediately before inversion: approximately 80 μm Immediately after inversion: approximately 60 μm

Relative size (volume) of the cells of the embryo during inversion	Reproductive cells are roughly 100 to 200 times larger than somatic cells [22]	Reproductive cells are roughly one to five times larger than somatic cells

Cell shape immediately before inversion	All cells are spindle shaped [20,22]	All cells are teardrop shaped

Cell shape in bend region during inversion	Flask-shaped cells (radially symmetrical at the chloroplast ends) [20,22,24]	Paddle-shaped cells (wedge shaped at the chloroplast ends)

Cell shape of cells that have passed the bend region	Column-shaped cells [20,22]	Pencil-shaped cells (the column shape does not appear until inversion is completed)

Order of cell shape changes	All cells undergo the same consecutive cell shape changes: spindle → flask → column [20,22,24]	Posterior and anterior hemispheres invert with different cell shapes changes; cells in the posterior hemisphere: teardrop → spindle → paddle (except for the cells close to the posterior pole) → pencil → column; cells in the anterior hemisphere: teardrop → disc → pencil → column

Shape of nuclei throughout inversion	Minor changes [20]	Major changes

Cytoplasmic bridges	Only between cells of the embryo [20,22,24,29]	Between cells of the embryo and between embryonic and parent cells

Phialopore	Swastika-shaped opening; four lips of the cell monolayer curl outward and backward [20,22]	Circular opening; while the opening widens, the cells and cytoplasmic bridges around the phialopore become passively stretched

Denting	Only before inversion [20,26,39]	Before and after inversion

Flagella	Only flagellar stubs approximately 2 μm in length that do not grow during inversion; flagella grow asymmetrically after inversion [76]	Flagella grow symmetrically throughout inversion

The presence of CBs between the cells of the embryo's cell monolayer and the coordinated movements of cells relative to the CB system appear to be essential for inversion in both *V. carteri *[20-22,24,27-29] and *V. globator*. Likewise, cell shapes and the order of coordinated cell shape changes are important for a successful inversion of embryos in both species. However, the order of the coordinated shape changes is different between the species, and the shapes also show differences. In *V. carteri *embryos, all cells are spindle shaped immediately before inversion [20,22]. *V. globator *embryos exhibit spindle-shaped cells only in the contracted posterior hemisphere, whereas cells in the anterior hemisphere are teardrop-shaped (Figure 12A, B and Table 1). The presence of flask-shaped cells in the bend region during inversion is characteristic of *V. carteri *embryos [20,22,24]. The long 'bottlenecks' of these cells, which localize at the chloroplast ends of the cells, are radially symmetrical. The paddle-shaped cells in the bend region of inverting *V. globator *embryos (Figure 12D) resemble the flask-shaped cells in the bend region of *V. carteri *embryos, in particular because both are arranged in the form of a spread fan. However, paddle-shaped cells are not radially symmetrical at their chloroplast ends but instead are wedge shaped (Figure 12D and Table 1), and they present no bottlenecks or stalks. Cells that have passed the bend region in *V. carteri *embryos are column shaped [20,22]. *V. globator *cells at the same developmental stage are pencil shaped (Figure 12E and Table 1), which means that these cells are more elongated along the chloroplast-end-to-flagellar-end axis than the corresponding cells in *V. carteri*. In *V. carteri *embryos, all (somatic) cells undergo the same consecutive cell shape changes from spindle to flask to column shaped [20,22]. In *V. globator *embryos, the posterior and anterior hemispheres invert associated with different cell shapes and a different order of shape changes. In the posterior hemisphere, embryonic cells change from teardrop to spindle to paddle (except for the cells close to the posterior pole) to pencil and then to column shaped. In contrast, cells from the anterior hemisphere change from teardrop to disc to pencil and then to column shaped (Figure 12).

In *V. globator*, cells appear to change their total volume during inversion, but it is unknown how this change occurs. Changes in volume might be achieved by emptying or filling contractile vacuoles; alternatively, cells might change their volume by exporting or importing cytoplasm to or from their neighbor cells through the CB system. In contrast, the cell volume is constant throughout inversion in *V. carteri *[27].

In a *V. carteri *embryo, inversion begins at a defined point at the anterior pole of the spheroid; the phialopore opens, and from this opening dramatic changes in cell shapes and coordinated movements of cells with respect to the CB system proceed from the anterior pole to the posterior pole [22] (Figure 1A). In contrast, a *V. globator *embryo shows a ring-shaped initiation of inversion somewhat below the equator of the spheroid. Changes in cell shape and coordinated movements of cells relative to the CBs start somewhat below the equator and proceed to the posterior pole; then a second process with changes in cell shape and coordinated movements of cells relative to the CBs begins at the former equator and proceeds to the anterior pole. In contrast to the *V. carteri *embryo, which starts inversion by opening the cellular monolayer, the *V. globator *embryo begins folding the cell layer without having a cell sheet with a free edge.

The phialopores in *V. carteri *and *V. globator *open in different ways. In *V. carteri*, a swastika-shaped opening appears, and the four lips of the cell monolayer curl outward and backward [20,22]. In *V. globator*, the opening is circular, and there is no curling of the cell layer. In contrast, the opening widens, while the anterior hemisphere moves over the posterior hemisphere (Figures 3F and 8B and Table 1).

In *V. carteri *(and two other *Volvox *species), the sporadic movements of the cell monolayer, referred to as denting, have only been observed immediately before inversion [20,26,39], while *V. globator *shows denting both shortly before and shortly after completion of inversion (Figures 6A and 10H, I and Table 1). Denting may be caused by non-simultaneous cell shape changes of individual cells or a patch of a few cells.

In summary, the comparison of type A and type B inverters suggests that both species follow the same basic principle: concerted, spatially and temporally coordinated changes in cellular shapes and concerted migration of cells relative to the CBs connecting them. However, differences exist in almost all details of the inversion process.

### Evolution of inversion

When the A and B types of inversion are mapped on the evolutionary tree of volvocine algae (see Additional File 1) [25,29,58,59,66,73,79-83], type B inversion seems to have been the earlier form of *Volvox *inversion because it appears on a deeper branch than type A inversion. However, if this hypothesis is true, type A inversion must have evolved independently in four different lineages from a type B ancestor [25,66]. If type A evolved before type B, it would mean that type B inversion evolved independently in three different lineages from a type A ancestor: in the section *Volvox *lineage, including *V. globator*, *V. rousseletii and V. barberi*, and in the lineages leading to *V. aureus *and to *V. dissipatrix*. Even if the repeated, independent evolution of type B from type A (or the other way round) is possible, the number of differences observed between type A and type B inverters makes it more reasonable that type A and type B inversion evolved in parallel. However, the true course of evolutionary events remains unclear. The different types of inversion and other differences observed during inversion described above substantiate a polyphyletic origin of the genus *Volvox*; this conclusion was also reached in earlier studies based on phylogenetic analyses of internal transcribed spacer sequences [63], sequences of five chloroplast genes [58,73] and comparison of flagellar beating patterns [83,84]. Thus, reclassification of this genus is required. A few species of this genus, including *V. globator*, form a small but robust monophyletic group, the section *Volvox*. The developmental and morphological differences of the species within the section *Volvox *compared to other *Volvox *species outside this section support the creation of a new genus for the species of the section *Volvox*.

### Biomechanical possibilities and implications about the involved tensile and compressive forces: a global mechanistic scenario

Epithelial folding during inversion is basically a biomechanical process whereby individual cells and cohorts of cells produce and respond to forces to generate the complex form of a developing multicellular organism [85]. Based on our results, which are summarized in Figures 12 and 13, and due to biomechanical possibilities and implications about the involved tensile and compressive forces, we developed the following global mechanistic scenario that predicts how a spherical cellular monolayer can turn itself inside out.

Before inversion in the spheroidal *V. globator *embryo begins, all cells are teardrop shaped (Figures 12A and 13A). This shape suggests that anisotropic cytoskeletal events along their chloroplast-end-to-flagellar-end axes formed these cells (Figure 12A1). The trigger for the beginning of the inversion process seems to be a so-far unknown signaling molecule or morphogen that does not appear to be uniformly distributed throughout the pre-inversion embryo (Figure 13A) but appears to be localized only, or at least mainly, in the posterior hemisphere of the embryo. Therefore, this putative trigger seems to operate only on the teardrop-shaped cells of the posterior hemisphere, which become increasingly spindle shaped (Figures 12B and 13B), while the cells of the anterior hemisphere remain teardrop shaped (Figures 12A and 13B).

The cross-section of spindle-shaped cells is smaller than the cross-section of teardrop-shaped cells (compare Figures 12A1 and 12B1), which may explain the contraction of the posterior hemisphere of the embryo (Figure 13B). This contraction occurs in conjunction with the formation of the bend region somewhat below the equator of the embryo (Figure 13B). Thus, the spherical embryo begins epithelial folding without having a cell sheet with a free edge (in contrast to the *V. carteri *embryo). In the bend region (Figure 13C), the spindle-shaped cells (Figure 12B) change more and more into paddle-shaped cells (Figure 12D). Simultaneously, these cells seem to move relative to the CBs so that the CBs of paddle-shaped cells eventually are at their chloroplast ends, which face the inner side of the curve (Figures 12D and 13C). The network of CBs appears to be the substrate upon which the coordinated cell shape changes operate to deform the cell sheet. The cell shape conversions together with the movements of cells relative to the CBs seem to progress from the equator of the embryo toward its posterior pole (compare Figure 13B to 13E), and appear to drive inversion of the posterior hemisphere and its simultaneous movement into the anterior hemisphere (Figure 13B, C, D, E, F).

However, how is the signal to change shape and CB positions forwarded to neighboring cells, and how is it coordinated? Once epithelial folding is initiated in the (circular) bend region, a radially symmetrical wave of a trigger molecule or simply of a mechanical force transmission might perpetuate and forward the folding command from cells in the bend region of the embryo toward the posterior pole of the embryo by inducing cell shape changes and movements of cells relative to the CBs in neighboring cells.

What shapes the paddle cells of the bend region? Fundamentally, the paddle-shaped cells (Figure 12D) in the bend region seem to be shaped by anisotropic cytoskeletal events along the chloroplast-end-to-flagellar-end axis (Figure 12D1), which produce strongly elongated cells that get thinner toward the chloroplast end (Figures 12D and 13C). Basically, these cells seem to be radially symmetrical throughout the length of the cell; thus, the cytoskeletal forces in radial directions of the cell seem to be isotropic. However, due to the increasing curvature of the bend region (especially in early inversion), the chloroplast ends of the cells come very close together, and a lack of space seems to arise at these ends of the cells. Finally, the cells appear to get compressed at the chloroplast end; thus, the minor axes of the cross-sections at these ends are only one-third or one-fourth the length of the major axes (Figure 12D3, D4). The smaller the radius of the bend region, the stronger the compression of the chloroplast end of the paddle-shaped cells appears to be. Therefore, the shape of the paddle cells at the chloroplast end (Figure 12D3, D4) seems to reflect an isotropic cytoskeletal activity in radial directions in the context of an anisotropic mechanical environment.

Once inversion of the posterior hemisphere is well advanced and the radius of the curvature in the bend region increases (Figure 13E), the paddle-shaped cells (Figure 12D) change into pencil-shaped cells (Figure 12E). The remaining spindle-shaped cells (Figure 12B) close to the not yet inverted posterior pole never become paddle shaped but directly change into the pencil shape (Figure 13E).

The invagination of the posterior cell sheet seems to generate circumferential tension along the rim of the anterior cap (Figure 13C, D). Therefore, the teardrop-shaped cells (Figure 12A) at the rim of the anterior cap (Figure 13C, D) become increasingly disc shaped (Figure 12C).

What shapes the disc cells? Basically, the disc-shaped cells (Figure 12C) might show a more or less isotropic cytoskeletal activity but, due to tensions on the cell sheet in the anterior-posterior direction of the embryo in response to pulling at the site of bending (Figure 13D), the cells get elongated and flattened (Figures 12C and 13D). Thus, disc-shaped cells seem to be shaped by an anisotropic mechanical environment. This anisotropic mechanical environment has a second consequence: the disc-shaped cells appear to begin to move over the rim of the anterior cap (Figure 13C, D), which seems to be the beginning of the inversion of the anterior hemisphere. The conversion of teardrop-shaped into elongated cells appears to progress in a radially symmetrical wave from the rim of the anterior cap of the embryo toward its anterior pole (compare Figure 13D and 13E). Eventually, tension in the anterior-posterior direction of the embryo produces thinning and elongation of the whole anterior cell layer (Figure 13D, E). It remains unclear whether the movement of the CBs to opposite ends of the disc-shaped cells (Figures 12C2 and 13E) is a passive response to pulling at the site of bending or whether there is an active remodeling of the cytoskeleton including active movement of the CBs; the latter seems more likely because embryonic cells can actively move the CBs [24].

Disc-shaped cells (Figure 12C) that moved over the rim of the anterior cap (Figure 13D, E) appear to condense into a smaller area beyond the rim, compared to the area they occupied before they rolled over the rim, that is, they reduce their cross-sections at the chloroplast ends and become pencil-shaped cells; simultaneously, these cells also seem to move relative to the CBs so that the CBs eventually are at their chloroplast ends (Figures 12E and 13E). Again, the network of CBs appears to be the substrate upon which the coordinated cell shape changes operate to act on the cell sheet. The conversions from disc to pencil shapes together with the concerted movements of the cells with respect to the CB system seems to maintain the circumferential tension on the rim of the remaining portion of the anterior cap and, as a consequence, on the not yet inverted cell sheet of the anterior hemisphere.

Because more and more disc-shaped cells (Figure 12C) move over the rim, the circumferential tension on the cell sheet of the anterior hemisphere increases continuously. When the remaining disc-shaped cells of the anterior hemisphere and their CBs are stretched to their maximum length, further tension on the anterior hemisphere results in the opening of the phialopore (Figure 13E).

The appearance of a circular opening means that the dominant tensions on the anterior hemisphere are likely to be circumferential. While the phialopore widens (Figure 13E, F, G), both the cells and the CBs around the phialopore as well as submarginal cells in the sheet and their CBs appear to become passively stretched along a circular line described by the edge of the opening (Figures 3F and 8B). However, the phialopore widens dramatically until the diameter of the opening is even greater than the outer diameter of the inverted posterior hemisphere (Figure 13F, G). This expansion of the phialopore cannot be explained only by stretching of the cell sheet; there also seems to be a rearrangement of cells at the phialopore.

The movement of disc-shaped cells (Figure 12C) over the rim of the remaining anterior cap and the conversion of disc-shaped cells into pencil-shaped cells (Figure 12E) continues; thus, the remaining anterior hemisphere appears to glide over the already inverted posterior hemisphere until the anterior cap is completely inverted (compare Figure 13E to 13H). This in-turning of the cell sheet over the surface of the outer layer is comparable to involution, one of the major morphogenetic movements that occur during gastrulation; involution can be observed, for example, in mesoderm migration in the Amphibian gastrula [86]. At the end of inversion, the phialopore gets smaller and smaller (Figure 13H). Relaxation of the previous circumferential tension that occurred during opening of the phialopore might be one reason but is probably not the only reason for this process, there also seems to be a rearrangement of cells at the opening. Finally, the phialopore closes without leaving any visible trace of the previous opening behind (Figure 13H, I).

Right after the completion of inversion, all cells of the embryo change concertedly from pencil (Figure 12E) to column shapes (Figures 12F and 13I). Somewhat later, they develop gradually into gemstone-shaped cells (Figure 12G). Following inversion, the juvenile organism resembles a miniature adult; it will increase in size (without further cell division) by depositing large quantities of ECM.

## Conclusions

Both type A and type B inverters must solve the same developmental problem: they begin their lives inside out and must turn their spherical cell monolayer outside in to achieve their adult configuration. Inverters of both types follow the same basic principle: concerted, spatially and temporally coordinated changes in cellular shapes act together with concerted migration of the cells relative to the CB system. However, differences exist in almost all details of the inversion process, suggesting analogous inversion processes that arose through parallel evolution.

Based on our results showing the cellular mechanisms of type B embryonic inversion in *V. globator *and due to biomechanical possibilities and implications about the involved tensile and compressive forces, we developed a global mechanistic scenario that predicts how a spherical cellular monolayer can turn itself inside out in *V. globator*. This mechanistic scenario might lay the groundwork for some interesting opportunities to learn how the cell biology, the biomechanical mechanisms of force generation, the dynamic regulation in space and time and the underlying signaling events are integrated such that they produce the global mechanical consequences driving epithelial folding.

## Methods

### Strain and culture conditions

The wild-type *Volvox globator *Linné strain SAG 199.80 was obtained from the Sammlung von Algenkulturen der Universität Göttingen (Culture Collection of Algae at the University of Göttingen, SAG), Germany [87]. Cultures were grown in standard *Volvox *medium (SVM) [88] at 22°C to 23°C under a cycle of 8 h dark/16 h light [89] with an average of approximately 100 μmol photons m^-2 ^s^-1 ^of photosynthetically active radiation. Small cultures were grown in a growth chamber in 10-mL glass tubes with caps that allow for gas exchange. Larger cultures were grown in a thermostat (COM 7865; Edwards, Kniese & Co., Marburg, Germany) in 1-L glass tubes aerated with sterile air at 150 cm^3 ^min^-1^.

### High-resolution *in vivo *stereo LM

High-resolution *in vivo *stereo LM was performed using a motorized and automated Leica MZ16A stereomicroscope (Leica Microsystems, Wetzlar, Germany) with fully apochromatic optics and a transmitted light illuminator with cold light sources [25]. In this system, a resolution of up to 840 Lp mm^-1 ^(= 0.6 μm) was obtained. For higher magnifications, an Axioskop 40 FL upright microscope (Carl Zeiss, Oberkochen, Germany) equipped with Achroplan, Fluar and Plan-Neofluar objectives of up to 100× magnification was used. A digital PowerShot S50 camera (Canon, Tokyo, Japan) with a 1/1.8" charge-coupled device sensor was used for photographic documentation.

### Microsurgery

For all SEM experiments, whole *V. globator *spheroids were fixed as described below. Using two tungsten needles (Plano, Wetzlar, Germany), the embryos were removed from their mother spheroids and were also removed from their embryonic vesicle under a stereo-microscope. In some experiments, embryos were subsequently fragmented using the tungsten needles. The tungsten needles were sharpened electrolytically [90]. For this step, a carbon electrode was placed into a saturated solution of potassium nitrate containing 10% potassium hydroxide. A tungsten needle was dipped into the solution (30×) while being subjected to low voltage (9.3 V) and alternating current.

### Fixation and dehydration of embryos

For LM of sections and for TEM and SEM, spheroids were fixed as described previously [20] with the following modifications. Fixation was performed in 5% glutaraldehyde (Agar Scientific, Stansted, UK) in SVM for approximately 16 h. Specimens were washed three times with 50 mM phosphate buffer (pH 7.0) for 10 min each and postfixed in 2% osmium tetroxide (Electron Microscopy Sciences, Hatfield, PA) for 1 h.

For LM of sections and for TEM, the fixed spheroids were dehydrated by passage (30 min each) through 30%, 60%, 90% and 100% (2×) ethanol.

For SEM, coverslips were coated with 0.1% polyethylene imine (PEI; Sigma-Aldrich, St. Louis, MO, USA), incubated at 20°C for 15 min, rinsed with deionized water and dried at 20°C. The PEI-coated coverslips were fractured, and a suspension of spheroids containing embryos (approximately 2 μL to 5 μL) was transferred onto a small fragment of a coverslip. The fixed embryos were removed from their mother spheroids and simultaneously from the surrounding embryonic vesicle, as described above. For some experiments, embryos were fragmented (see above). Intact and fragmented embryos were allowed to settle on the same piece of PEI-coated coverslip. The embryos that settled on the coverslip fragment were dehydrated by passage through increasing concentrations of ethanol (see above) and then critical point dried using a Bal-Tec CPD 030 critical point dryer (Leica Microsystems).

For cLSM, intact spheroids were fixed as described previously [72]. Samples were maintained under gentle motion throughout the fixation period using a LD-79 test-tube rotator (Labinco, Breda, Netherlands) at 5 rpm. Spheroids were fixed for 15 min in 3.7% formaldehyde, 0.1 mM dithiothreitol (DTT), 0.1% Triton X-100, 2 mM magnesium chloride, 5 mM ethylene glycol-bis(2-aminoethylether)-N, N, N', N'-tetraacetic acid (EGTA), 150 mM potassium chloride, 10 mM sodium glycerophosphate and 10 mM 4-(2-hydroxyethyl)-1-piperazineethanesulfonic acid (pH 7.0), washed three times for 2 min with 0.1% Tween in phosphate-buffered saline (TPBS) and incubated for 4 h in 1% (tergitol-type) nonyl phenoxypolyethoxylethanol, 1 mM DTT, 1% BSA, 2 mM magnesium chloride, 5 mM EGTA, 150 mM potassium chloride and 10 mM sodium glycerophosphate to extract chlorophyll and minimize autofluorescence. Finally, specimens were washed three times for 5 min each in TPBS.

### Embedding and sectioning

For LM of sections and for TEM, the dehydrated specimens were infiltrated in a 1:1 solution of TAAB transmit resin (Agar Scientific) to ethanol (v/v) for 1 h, followed by a 2:1 solution of TAAB transmit resin to ethanol (v/v) for approximately 16 h. After evaporation of the ethanol, specimens were infiltrated twice in pure TAAB transmit resin in an evacuated dessicator for 2 h each. Specimens in resin were transferred to BEEM capsules (Agar Scientific), and the resin was then polymerized through a 16-h exposure at 70°C. The polymerized blocks were removed from the BEEM capsules, and most of the excess resin was trimmed away with a razor blade under a stereo-microscope.

For LM, sections with a thickness of approximately 2 μm were cut with glass knives on a Reichert-Jung Ultracut E ultramicrotome (Reichert Microscope Services, Depew, NY, USA) and transferred to glass slides.

For TEM, sections with a thickness of approximately 80 nm were cut with a diamond knife (6620 SU; Micro Star Diamond Knives, Huntsville, TX, USA) on the Ultracut E ultramicrotome and mounted on 400 mesh copper grids (Plano).

### Staining

Sections on glass slides (approximately 2 μm in thickness) were stained for LM by incubating the slides in 0.1% toluidine blue (Sigma-Aldrich) for 10 s on a Combimag RCT hot plate stirrer (IKA-Werke, Staufen, Germany) at 70°C. Thereafter, the slides were rinsed in deionized water; the sections were covered with droplets of deionized water; and the slides were placed on the hot plate stirrer until the water had evaporated. The sections were then mounted in Entellan rapid mounting medium (Merck, Darmstadt, Germany).

Sections for TEM (approximately 80 nm in thickness) on grids were stained with 0.1% uranyl acetate (Plano) for 10 s, washed by dipping them in deionized water, stained with 2% lead citrate (Agar Scientific) for 10 s, and washed again with deionized water.

Intact, fixed spheroids for cLSM analysis were stained with 38 μM phalloidin-TRITC (Sigma-Aldrich) in TPBS for 3 h.

Phalloidin-TRITC stains F-actin specifically [72]. In our experiments, phalloidin-TRITC was used primarily as a cytoplasmic stain. While the resolution of the cLSM (Leica TCS SP2; Leica Microsystems) is insufficient to see individual actin filaments, a rough intracellular localization of F-actin is feasible. After phalloidin-TRITC staining, 4 μg/mL 4',6-diamidino-2-phenylindole dihydrochloride (DAPI; Sigma-Aldrich) was added to visualize the nuclei, and incubation continued for 1 h. The spheroids were washed three times in TPBS and allowed to settle on PEI-coated coverslips. Attached spheroids were mounted in 50% glycerol (Merck) in PBS. The specificity of the phalloidin staining was confirmed by comparison of embryos treated only with TRITC-labeled phalloidin with embryos treated with TRITC-labeled phalloidin plus unlabeled phalloidin.

### Sputter coating

For SEM, critical point-dried specimens were coated with gold in a Bal-Tec SCD 005 cool sputter coating system (Leica Microsystems). The working distance was 50 mm; the current was 30 mA; the argon sputtering pressure was 5 Pa; and the sputtering time was 300 s. The thickness of the gold layer was approximately 35 nm.

### LM of sections

Toluidine blue-stained sections (approximately 2 μm in thickness) of embryos were analyzed with an Axioskop 40 FL upright microscope (Carl Zeiss) using oil immersion objectives.

### TEM

Heavy metal-stained sections (approximately 80 nm) were examined with a Hitachi H-500 TEM (Hitachi, Tokyo, Japan) operated at 75 kV and with a Philips CM-100 TEM (Philips, Eindhoven, Netherlands) operated at 80 kV.

### SEM

Gold sputter-coated specimens were analyzed with a Hitachi S-450 SEM (Hitachi) operated at 15 kV.

### cLSM

Fixed and stained specimens were examined with an upright Leica TCS SP2 cLSM (Leica Microsystems). DAPI was excited at 405 nm, and the emitted blue fluorescence was detected with a band-pass filter at 430 nm to 490 nm. TRITC was excited at 543 nm, and the emitted red fluorescence was detected with a band-pass filter at 580 nm to 650 nm.

### Two-dimensional and 3D modeling

LM, cLSM, SEM and TEM images of intact and sectioned embryos before, during and after inversion were adjusted to the same magnification. In this adjustment, shrinkage during processing (for example, through fixation and dehydration) was taken into account and corrected with the help of images of live material. Adjusted images were screened for the anatomical characteristics of interest and all appropriate images with a non-ambiguous assignment to a certain area of the cell monolayer relative to the anterior and posterior pole of the embryo were selected for further analysis. In these images, contours of cells and nuclei, the position of CBs relative to the cell and the curvature of the cell monolayer were plotted in separate image layers using Photoshop CS5 software (Adobe Systems, San Jose, CA, USA). Contours of cells were assigned to the categories teardrop, spindle, disc, paddle, pencil, column and gemstone shaped. Dimensions (for example, diameters and lengths) and arithmetical means of all dimensions of anatomical characteristics were determined. At least 30 contours of the same anatomical structures were laid on top of the others to develop two-dimensional (2D) and 3D consensus shapes. 3D wireframe modeling and texturing was performed using Blender (version 2.49b) software (Stichting Blender, Amsterdam, Netherlands). 2D vector graphics were developed using CorelDRAW Graphics Suite X5 (version 15) software (Corel, Ottawa, Canada).

## Abbreviations

2D: two-dimensional; 3D: three-dimensional; BSA: bovine serum albumin; CB: cytoplasmic bridge; cLSM: confocal laser scanning microscopy/microscope; CZ: cellular zone; DAPI: 4',6-diamidino-2-phenylindole dihydrochloride; DTT: dithiothreitol; ECM: extracellular matrix; EGTA: ethylene glycol-bis(2-aminoethylether)-N, N, N',N'-tetraacetic acid; F-actin: filamentous actin; LM: light microscopy/microscope; PBS: phosphate buffered saline; PEI: polyethylene imine; SEM: scanning electron microscopy/microscope; SVM: standard *Volvox *medium; TEM: transmission electron microscopy/microscope; TPBS: Tween in phosphate-buffered saline; TRITC: tetramethylrhodamine B isothiocyanate.

## Authors' contributions

SH conducted the experiments and analyzed the data. AH conceived and coordinated the study, critically evaluated the data, and wrote the manuscript. Both authors read and approved the final manuscript.

## Supplementary Material

Additional file 1 **Evolutionary tree of volvocine algae**. An evolutionary tree of volvocine algae based on the nucleotide sequences of five chloroplast genes. This phylogenetic analysis indicates that multicellularity evolved only once in this group. There are two fundamentally different sequences through which embryos of the genus *Volvox *turn right-side out: type A and type B inversion. The type of inversion is indicated for all species of the genus *Volvox*. Click here for file
