# Supplementary material for: There is more than one way to turn a spherical cellular monolayer inside out: type B embryo inversion in Volvox globator
Source: BMC Biol. 2011 Dec 29;9:89. doi: 10.1186/1741-7007-9-89 (PMC3324393; doi:10.1186/1741-7007-9-89)
Supplement: Additional file 1 — Evolutionary tree of volvocine algae. An evolutionary tree of volvocine algae based on the nucleotide sequences of five chloroplast genes. This phylogenetic analysis indicates that multicellularity evolved only once in this group. There are two fundamentally different sequences through which embryos of the genus Volvox turn right-side out: type A and type B inversion. The type of inversion is indicated for all species of the genus Volvox. [file 1741-7007-9-89-S1.PDF]

Evolutionary tree of volvocine algae

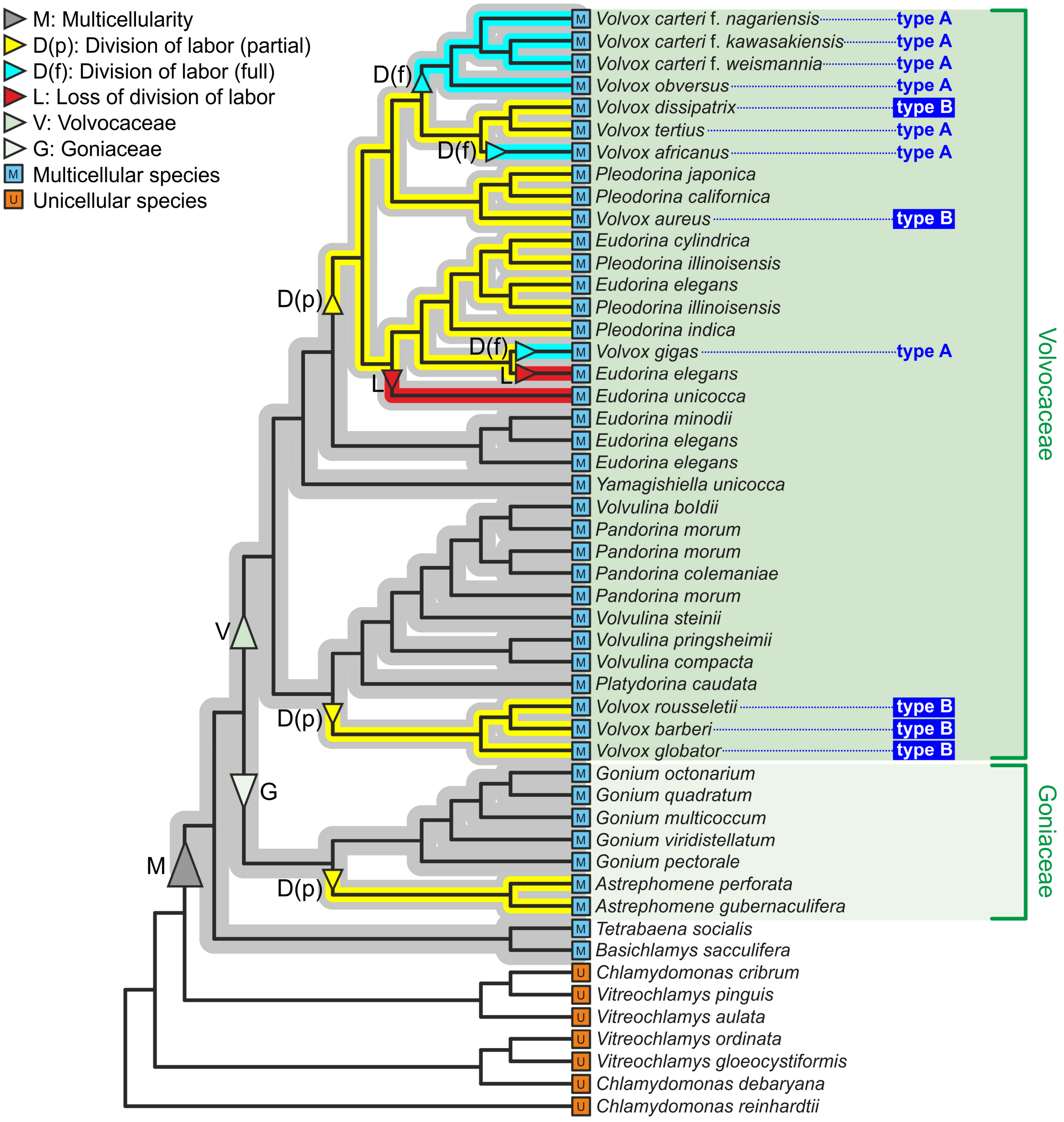

An evolutionary tree of volvocine algae based on the nucleotide sequences of five chloroplast genes. This phylogenetic analysis indicates that multicellularity evolved only once in this group. In contrast, a partial germ-soma division of labor evolved independently in three different lineages and was lost twice (Nozaki et al. 1997; Hoops et al., 2005; Herron and Michod 2008; Herron et al. 2009; Hallmann 2011). A full

germ-soma division also evolved three times. There are two fundamentally different sequences through which embryos of the genus *Volvox* turn right-side out: type A and type B inversion (Nozaki, 2003; Hallmann, 2006). The type of inversion is indicated in blue for all species of the genus *Volvox*. The meanings of symbols and letters are given in the figure. This tree was adapted from Herron and Michod (2008) and others (Sachs 2008; Herron et al., 2009; Herron et al. 2010; Hallmann, 2011); some additional information was added (Hallmann, 2006; Lerche and Hallmann 2009; Ueki et al. 2010).

References

- Hallmann A: **Morphogenesis in the family Volvocaceae: different tactics for turning an embryo right-side out**. *Protist* 2006, **157**:445-461.
- Hallmann A: **Evolution of reproductive development in the volvocine algae**. *Sex Plant Reprod* 2011, **24**:97-112.
- Herron MD, Desnitskiy AG, Michod RE: **Evolution of developmental programs in *Volvox* (Chlorophyta)**. *J Phycol* 2010, **46**:316-324.
- Herron MD, Hackett JD, Aylward FO, Michod RE: **Triassic origin and early radiation of multicellular volvocine algae**. *Proc Natl Acad Sci USA* 2009, **106**:3254-3258.
- Herron MD, Michod RE: **Evolution of complexity in the volvocine algae: transitions in individuality through Darwin's eye**. *Evolution* 2008, **62**:436–451.
- Hoops HJ, Nishii I, Kirk DL: **Cytoplasmic bridges in *Volvox* and its relatives**. In: *Cell-Cell Channels.* Edited by Baluska F, Volkmann D, Barlow PW. Georgetown, Texas: Eurekah.com; 2005: 1-20.
- Lerche K, Hallmann A: **Stable nuclear transformation of *Gonium pectorale***. *BMC Biotechnol* 2009, **9**:64.
- Nozaki H: **Origin and evolution of the genera *Pleodorina* and *Volvox* (Volvocales)**. *Biologia (Bratisl)* 2003, **58**:425-431.
- Nozaki H, Ito M, Uchida H, Watanabe MM, Kuroiwa T: **Phylogenetic analysis of *Eudorina* species (Volvocaceae, Chlorophyta) based on *rbc*L gene sequences**. *J Phycol* 1997, **33**:859-863.
- Sachs JL: **Resolving the first steps to multicellularity**. *Trends Ecol Evol* 2008, **23**:245-248.
- Ueki N, Matsunaga S, Inouye I, Hallmann A: **How 5000 independent rowers coordinate their strokes in order to row into the sunlight: Phototaxis in the multicellular green alga *Volvox***. *BMC Biol* 2010, **8**:103.
